# Building research capacity and culture: Exploring nurses' experience of implementing a nurse‐led clinical trial

**DOI:** 10.1111/jonm.13576

**Published:** 2022-03-15

**Authors:** Catherine O'Brien, Eileen Furlong, Barbara Coughlan, Patricia Fox, Andrew Darley

**Affiliations:** ^1^ The Haematology, Oncology and Palliative Care (HOPe) Directorate St James's Hospital Dublin Ireland; ^2^ School of Nursing Midwifery and Health Systems, Health Sciences Centre University College Dublin Dublin Belfield Ireland; ^3^ School of Medicine, Health Sciences Centre University College Dublin Dublin Belfield Ireland

**Keywords:** facilitators, nurse experience, qualitative research, transformational leadership

## Abstract

**Aim:**

To explore the experiences of a nursing team who implemented an international nurse‐led clinical trial in practice and understand the facilitators to their involvement.

**Background:**

The role and responsibilities of the clinical nurse are advancing to encompass research activity to help inform evidence‐based practice. However, several personal and organisational challenges can inhibit nurses' capacity to implement and undertake research within clinical practice.

**Methods:**

Three focus groups were conducted with members of a nursing team (*N* = 18). Thematic analysis was employed, and themes were identified and agreed upon by the research team.

**Results:**

Five themes were identified: ‘Previous experience of and attitudes to participation in clinical research’, ‘Decision‐making regarding participation in the clinical trial’, ‘Facilitators of participation in the clinical trial’, ‘Challenges of research in nursing practice’ and ‘Future orientation towards research’.

**Conclusion:**

Through their experiences of implementing a nurse‐led clinical trial within practice, nurses recognized a number of facilitators and challenges to their participation. The perceived relevance of the clinical trial to the nurses' practice, potential to improve patient care and appreciation of the nurse leader's expertise and understanding of their context were key motivators. Reciprocal trust with the nurse leader who was encouraging, motivating, supportive and accessible resulted in the engagement and commitment of the nursing team.

**Implications for Nursing Management:**

This paper offers a perspective that can inform senior nursing management teams when implementing and conducting evidence‐based research amongst nursing teams and in doing so meet the needs of developing research capacity amongst clinical nurses.

## 1 BACKGROUND

The role and responsibilities of the clinical nurse are ever‐evolving and transforming, whereby today the practice of research is a key competency of the role (DeNisco & Barker, 2013). Nursing has become a distinctive scientific discipline, which requires its own body of knowledge to facilitate evidence‐based practice (Chen et al., 2019). Opportunities to engage in research, however, are perceived to be lacking in the clinical practice setting (Hagan, 2018). A key focus of the literature to date has centred on how nurses implement research evidence into their practice (Heydari et al., 2014; Keib et al., 2017; Leach & Tucker, 2018; Ryder & Jacob, 2021). While evidence exists regarding nurses implementing and undertaking research, studies commonly focus on the barriers to their research engagement. Key challenges include personal factors such as lack of knowledge or training, negative attitudes and lack of opportunities (Chien et al., 2013; Lode et al., 2015; Timmins et al., 2012; Vijayalakshmi et al., 2014) and organisational factors include lack of resources, time, funding and support from fellow clinicians (Hagan & Walden, 2017; Lode et al., 2015; Sanjari et al., 2015; Silka et al., 2012). With the expectation for clinical nurses to be research active, there is a need to build research capacity and support nurses in all aspects of the research process (Birkhoff et al., 2020; Cooper et al., 2021; McKee et al., 2017).

Research capacity refers to the ability to conduct nursing research activities in a sustainable manner in a specific context, normally at a group rather than individual level (Chen et al., 2019), while initiatives to support nurses to acquire research skills are necessary. Several components have been identified as crucial to building research capacity: competence, motivation, infrastructure including material and management support, academic/clinical collaboration and strong leadership (Cooke et al., 2018; Cooper et al., 2021; McKee et al., 2017). The context of an international, multicentre, nurse‐led clinical trial was recently used as an opportunity to build research capacity within a cancer nursing team. The aim of this study was to explore the experiences of a nursing team who implemented an international nurse‐led clinical trial in order to understand the facilitators to their involvement in the study.

## 2 METHOD

### 2.1 Study design

This was a qualitative study using Thematic Analysis, a pragmatic approach to qualitative research commonly applied within health care research (Braun & Clarke, 2021). The study was guided by the three key research questions:

- What are the experiences of nurses who implemented a nurse‐led clinical trial within their practice?
- What motivators exist for nurses to engage in conducting research?
- What are the facilitators and challenges of building research capacity and research culture within a nursing team?

### 2.2 Context of the clinical trial

The eSMART® clinical trial tested the advanced symptom management system (ASyMS) intervention which utilizes mobile device technology to enable real‐time, 24‐h monitoring and management of patients' self‐reported chemotherapy‐related symptoms within a European multi‐centre context (Furlong et al., 2019; Maguire et al., 2021). Patients with cancer used a dedicated mobile device to complete a chemotherapy‐related symptom questionnaire daily. For symptoms that required clinical intervention, the algorithm generated ‘real time’ alerts to the clinical sites via a dedicated clinician handset and alerts were managed by the nursing team (‘alert handlers’) 24‐h a day, 7 days a week. The eSMART trial was conducted in the cancer centre over a four‐year period, including a randomized controlled trial that began in July 2016 and was completed in December 2018.

The Lead Cancer Nurse, who was responsible for implementing the study, that is, nurse leader (NL), had worked in a clinical capacity at the cancer centre for over 15 years. The nursing team who participated in the clinical trial comprised 64 nurses from various nursing roles, including staff nurses, nurse managers and clinical nurse specialists, who were based in the oncology dayward and in‐patient oncology ward. Staff participation in the clinical trial was on a voluntary basis and was not a requirement within their role. The NL was responsible for staff training, patient recruitment, management of study equipment, data completion, reporting technical/clinical issues and alert handling. The clinical nurses were responsible for handling alerts generated by patients.

### 2.3 Participants

Purposive sampling was employed to recruit participants (i.e., members of the nursing team involved in the clinical trial) who would provide a comprehensive and well‐developed understanding of the phenomenon of interest (Liamputtong & Ezzy, 2005). To be eligible, nurses had to be currently employed at the cancer centre and had to have participated in the implementation and undertaking of the clinical trial at any stage of its duration. The study was advertised by poster and requested nurses to contact the research team if they wished to participate.

### 2.4 Data collection

Three focus groups (FG) were conducted with a total of 18 participants who met the inclusion criteria once the clinical trial was completed. This data collection method was chosen to draw on the group interaction to generate insights that may not have been possible without the interaction within the group (Barbour & Morgan, 2017). FG were conducted in a private boardroom within the cancer centre at a time that did not interfere with the participants' working day. FG varied in size as follows: FG1 (*n* = 4), FG2 (*n* = 8) and FG3 (*n* = 6) respectively. Each FG was conducted by two facilitators, who did not have a working relationship with the participants. Two of the facilitators were members of the clinical trial research team within an academic institution, while the third was impartial had no involvement in the clinical trial. A semi‐structured topic guide was designed for the FGs. FG duration ranged between 31 and 52 min. A short demographic information form was given to each participant to complete at the end of the FG to capture their educational background and professional experience, as detailed in Table 1.

**TABLE 1:** Participant demographics

Characteristic	Participants (*n* = 18)
Gender	Female: *n* = 18 (100%)
	Male: *n* = 0 (0%)
Educational qualification	Graduate level: *n* = 3 (17%)
	Postgraduate level: *n* = 15 (83%)
Years of experience in cancer nursing	2–4 years: *n* = 3 (16.6%)
	5–9 years: *n* = 6 (33.3%)
	10–14 years: *n* = 3 (16.6%)
	15–19 years: *n* = 6 (33.3%)
Nursing role	Staff nurse: *n* = 7 (39%)
	Nurse manager: *n* = 6 (33%)
	Clinical nurse specialist: *n* = 5 (28%)

Field notes were written after each focus group. During data collection, the research team discussed emerging themes to ensure the topic guide reflected the context and was refined if necessary. Data collection was complete when no new themes emerged.

### 2.5 Data analysis

FG interviews were digitally recorded and transcribed verbatim. Thematic analysis was used to analyse the data which enabled the research team to identify, interpret and describe in rich detail key elements of participants' accounts and to organise them into meaningful themes systematically and reflexively (Braun & Clarke, 2021). This analysis process included six steps, as defined by the authors: (i) becoming familiar with the data and note taking, (ii) systematically coding the data, (iii) generating initial themes from the coded data, (iv) developing and reviewing themes based, (v) refining, defining and naming themes and (vi) writing the report. One author conducted the initial five steps using Microsoft Word documents and subsequently presented the preliminary themes to the research team. All members of the research team reviewed the proposed themes and read the original data transcripts to validate, refine and agree on the final study themes. To enhance the trustworthiness of the study (Lincoln & Guba, 1985), member checks were undertaken. Participants were sent the final version of the findings and provided feedback for comprehensiveness, with no changes suggested. The research was guided by the Consolidated Criteria for Reporting Qualitative Research (COREQ) (Tong et al., 2007).

### 2.6 Ethical considerations

Participants received written information about the study and were assured confidentiality and their right to withdraw, without any consequences. Written informed consent was obtained from each participant prior to FG commencement. All personal data was pseudo‐anonymized and an identification number was assigned to each participant. The code list was stored in a secure file password‐protected Microsoft Word document, which was only available to the research team.

## 3 FINDINGS

**FIGURE 1:**
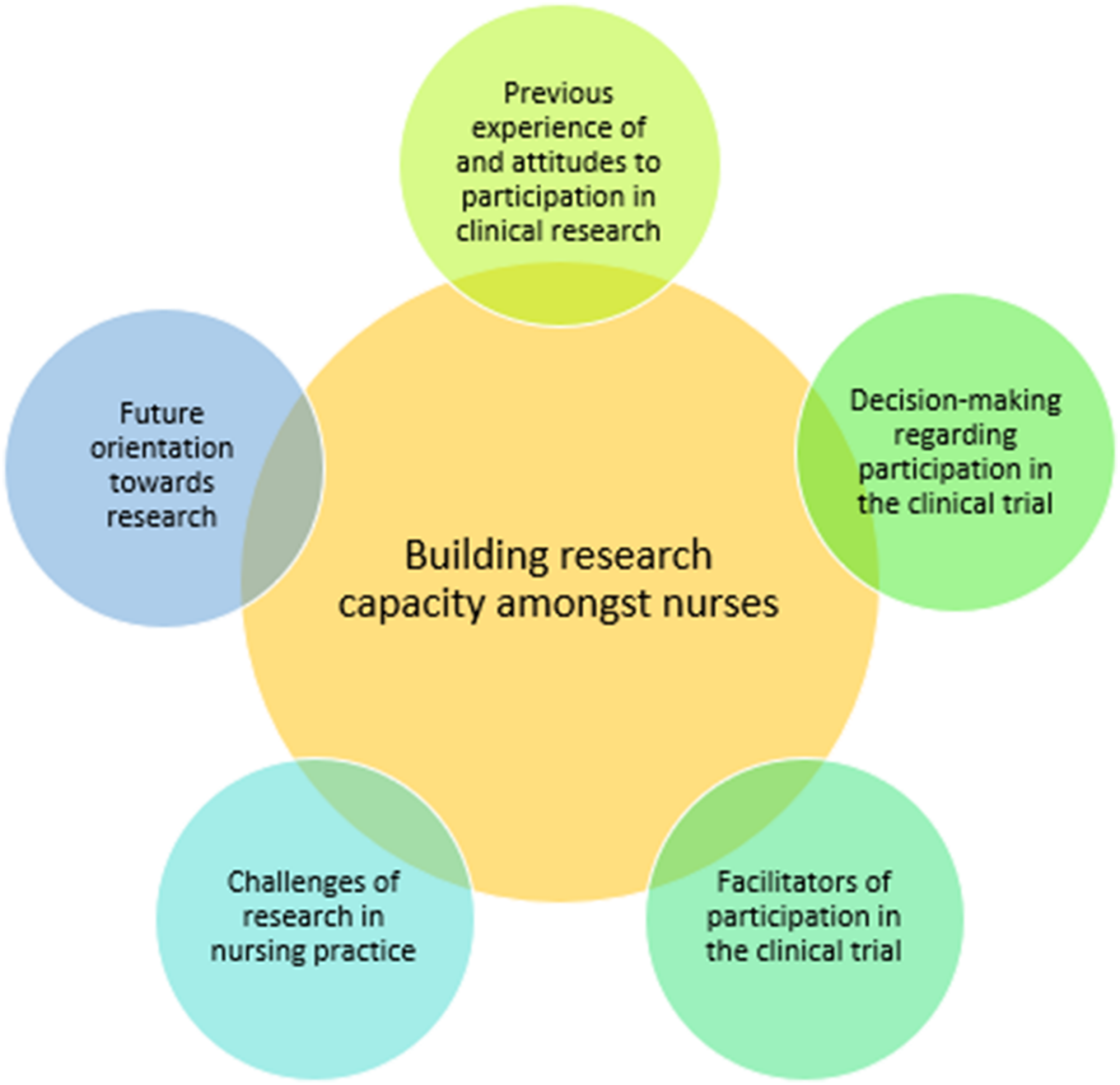
Identified themes

Thematic analysis of the FG transcripts identified five major themes, as outlined in Figure 1.

The following findings represent a variety of perspectives of the participants who took part in the clinical trial and their experience of undertaking research. Participant quotations are utilized to illustrate the themes and to highlight the consistency between the data and the research team's findings (Eldh et al., 2020).

### 3.1 Previous experience of and attitudes to participation in clinical research

While all participants had achieved a third‐level graduate or post‐graduate qualification in cancer nursing, most participants reported having no formal research training in clinical research. A small number of participants reported their experience of writing research proposals, conducting literature reviews as part of their academic training or being part of a clinical audit at their organisation. Most participants reported having limited practical experience with research. Although participants were aware of clinical trials being conducted at their cancer centre, they commented on how they ‘*would not really be part of it*’ and that there were not ‘*too many opportunities*’ for nursing staff. Research was viewed as being unfeasible in their role due to the existing duties in patient care, staffing grade and resources available to them:

*‘I think it's very difficult for anyone on the frontline, staff nurse, CNS, CNM to expect them to incorporate a huge amount of research into their working life. It's already under‐resourced and short‐staffed, you know? The main thing is that the patient is looked after in front of you … it's unrealistic unless we get a huge amount of resources to support that’*. (FG1)

Participants also reflected on their lack of research experience and questioned whether it was due to the lack of opportunities available to them or the lack of assertiveness within nursing culture to initiate research studies:

*‘I suppose as a group we are not very good and proactive at doing research. I think it's slowly improving; we all talk about how important it is’*. (FG1)

Some viewed research as being predominantly medically led whereby nurses' involvement pertained to assisting with administrative aspects such as recruitment rather than being a member of a research team. Regardless, participants recognized the importance of research to improve clinical practice while acknowledging nurses' potential as researchers: ‘*I think we all around the table have very good ideas about research but it's about getting it across the line*’ (FG2).

### 3.2 Decision‐making regarding participation in the clinical trial

Participants discussed their experience of deciding to take part in the clinical trial when it was presented to them by the NL. A key motivator was how they recognized the research study as an international project being led by nurses, which they believed was unique and engaging and could improve their patient outcomes:

*‘It's only by doing these audits and research that practice is going to change, to be part of that* …’ (FG2)

Participants commented on their awareness of how the clinical trial was being embedded in their standard patient care practices and though their participation was on a voluntary basis, some felt that it would be beneficial to support the project rather than excluding themselves from it:

*‘It was going nowhere; we were not getting away without doing it! (Group laughs) … we had a choice obviously but also it was put to us that it was going to be for the long‐term benefit of the service’*. (FG1)

Similarly, one participant who had changed roles within the nursing team after the clinical trial had commenced, commented on their perception of the research culture that was established in their clinical practice:

*‘I had moved up to the dayward in the middle of it so everyone had their education and everything, so I think it was like a ward culture thing that I had got in on it because everyone else pretty much had as well’*. (FG2)

Conversely, some participants expressed apprehension and anxiety about participating due to their current responsibilities and perception that the research was an additional workload that they would have to navigate:

‘*I thought it was beneficial to the nursing itself. And I thought it was a great idea. Great initiative for the patients. I remember, when initially, when we started it, you know the first month or so, there were things like, you know, is this really needed? And is this extra work?’* (FG3)

### 3.3 Facilitators of participation in the clinical trial

Leadership was identified by participants as a central facilitator in their implementation of the clinical trial and undertaking its related responsibilities. Although the study design and protocol were developed by a European Consortium (Maguire et al., 2021), participants stated that the NL was the ‘*big driver*’ or ‘*driving force*’ of the clinical trial as she articulated the relevance of the study to the nurses' everyday clinical practice. Participants remarked on the NL's enthusiasm and passion about the clinical trial which, in turn, enhanced their enthusiasm:

*‘She was definitely enthusiastic from day one and that definitely translated through the whole team’* (FG1)

Participants observed that the NL cultivated a supportive environment which made the research feasible and motivated them to engage. Several participants appreciated how the NL made the research ‘*visible*’ to them in the clinical environment and study‐related materials were easily available. It was noted that the NL maintained consistent and transparent communication with the team regarding the nature of the research study and its progress:

*‘(NL) is really good at being persistent and very present. Isn't she?’* (FG3)

*‘(NL) would always let you know how many you had recruited and how many were to go’* (FG1)

Participants discussed how the research environment was supported by the fact that the NL was a member of their cancer nursing team and known to all the staff participating in the clinical trial therefore understood the context in which they were working. One participant recognized the NL's intuition about their concerns:

*‘[NL] was a very strong confident leader and she very much outlined the support network that was there, so I think she … anticipated our hesitancy about it. So having someone that knows us and knows the clinical area was a huge bonus to us’*. (FG2)

Participants' perception of the NL's understanding of their working environment and responsibilities facilitated a sense of trust with her from the beginning of the project, as well as their own recognition of their abilities to implement and undertake the research. Participants appeared to develop a confidence in themselves arising from the NL's belief in their abilities:

*‘When you are a staff nurse, everything comes to promotional grade, so it makes it more attainable and doable’*. (FG1)

Participants also recognized the NL's commitment to the clinical trial, unwavering belief in the study and her availability and support which they found inspiring and motivating:

*‘I remember one of the students ringing her at the weekend and she decided to come over, like she was off that day, and she came in to show her exactly because she was not able to explain it over the phone so you know she was there’* (FG3)

Second, a key facilitator was the shared accountability and support network they established to undertake the clinical trial:

‘*You always knew who to go to if you were unsure or whatever, because so many people had decided to participate, there was lots of people to chat around it. There was a good team effort I think’*. (FG2)

Following official research training on recruitment and data collection for the study provided by the NL and the research team at the initiation of the study, participants described how they also learned from each other regardless of staff role or nursing experience‐level: *‘I think I probably learned more from the girls who were already doing it’* (FG2). Additionally, the NL established a WhatsApp group, including staff involved in the study, to maintain a dialogue and answer any queries: ‘*the option was there to ask your peers*’ (FG2). Third, the perceived benefits of undertaking the clinical trial, focusing on their clinical practice, was considered as a motivational facilitator. Participants had a sense of ownership regarding the research as it was nurse‐led and regularly referred to the study as ‘*ours’*. Nurses recognized that they were contributing to cancer care practice both on a local and international level which could have future benefits for patient care. Participants also mentioned the benefit of funding received for participating in the trial which was used to invest in their further education or conferences. One participant mentioned how they included this research experience during a promotional job interview.

### 3.4 Challenges of research in nursing practice

Several challenges for the nursing team existed at the time of participating in the clinical trial including the refurbishment and expansion of the inpatient oncology ward, a high level of staff turnover and poor skill mix of staff members. One participant encapsulated how stressful this period was for them as a team and questioned how the trial remained successful in its execution:

‘*Speaking from the inpatient side of things we were definitely in a turbulent period of time on the ward, it was very busy, very acute, very junior, no level of skill mix and considering all of that this was still successful and for there to be the element of commitment there was to it, does not really make sense*?’ (FG2)

Nurses who were members of the night staff felt that *‘there wasn't as much support from managers or senior staff*’ in comparison to team members working on the dayward area. Several participants commented on the difficulty they experienced in negotiating their responsibilities with the requirements of the clinical trial:

‘*You're trying to prioritize your workload and you have things that are equal priority on the ward and then you are trying to kind've reprioritize and manage that. There were challenges like that on the inpatient ward weekly for sure*’. (FG2)

Additionally, some participants commented on how they believed there was excessive responsibility and pressure placed upon the NL in coordinating the research study. One participant underlined how the duties of the NL were ‘*too much for one person*’ and others commented that it would have been beneficial if the responsibilities were shared with other designated leaders.

### 3.5 Future orientation towards research

A resounding belief was evident in participants' accounts of how they now view undertaking clinical research as accessible and achievable in conjunction with their designated nursing duties: ‘*it does not have to feel like an extra job*’. Several participants reported their awareness and understanding of research concepts because of participating in the clinical trial including ethics, study implementation, participant recruitment, protocol adherence and documentation. Participants commented on the research ‘*ethos*’ that has now been initiated amongst their team. Although participants recognized that research was achievable within their current role and how the NL ‘*got it across the line*’, some participants emphasized the importance of shared responsibility and to ensure that accountability is delegated to more than one person in their team in the future:

‘*She* [NL] *handled everything, and even when she changed her roles, she still took it on. I think that is one big challenge … everyone is changing their jobs and that could've had a negative impact on the study. So maybe having one or two, maybe two or three people, taking, leading the research, especially when the research is such a big study*’ (FG3)

## 4 DISCUSSION

The findings of the current study shed light on the experiences of a nursing team undertaking and integrating a clinical trial within their practice. Echoing previous evidence (Hagan, 2018; Lode et al., 2015; McKee et al., 2017), our study found that prior to their involvement in the clinical trial, the nurses believed undertaking clinical research was not within their remit and they did not expect to be involved (Van Oostveen et al., 2017) and previous experience was based on the administrative aspects of medical clinical trials. While participants recognized the value of research to nursing practice (Timmins et al., 2012), reservations existed at the beginning of the clinical trial regarding undertaking the research within their role, however, all participants joined the research team on a voluntary basis after being invited by the NL.

While culture and restraints within nursing practice are not conducive to research practice (Birkhoff et al., 2020), context was a critical factor in determining nurses' interest and motivation to implement and undertake the clinical trial. First, nurses recognized how the aim of the nurse‐led clinical trial was relevant to their role, whereby it has been found that when research is not relevant to nursing practice it can be a barrier (Hagan & Walden, 2017; McKee et al., 2017). Participants identified how the study aligned with their practice and how it may ultimately improve patient care and identify improved care methods. Second, nurses recognized that the NL understood what it meant to be a cancer nurse, their responsibilities and the nature of the patient profile. Participants were aware of the NL's expertise and understanding of their environment which, in turn, inspired and fostered the belief that they were capable of conducting clinical research within their role. This is outlined in previous literature as imperative to effective leadership (Collins et al., 2020; Ferreira et al., 2020). Consequently, the team further built on their trusting relationship with the NL, previously identified as being important for implementing change (Doody & Doody, 2012; Xu, 2017), and was a key motivator to their involvement in the clinical trial.

Acknowledging the longitudinal nature of the clinical trial, nurses cited how the funding received to implement the research was a motivational factor during the period as they recognized how it was being re‐invested into the nurses' education and research or professional development. This is supported by Lode et al.'s (2015) systematic review who highlighted how funding was pivotal to increasing research capacity amongst nurses. Consistent with Birkhoff et al.'s (2020) findings, nurses in this study felt valued for their contribution to an international, multicentre clinical trial which had the potential to improve clinical practice. They expressed ownership of the clinical trial and believed that it was a study conducted *by them* in which results may improve nursing practice *for them*. Thus, participants understood that while they may not be named in the clinical trial publications, they valued their contribution in making the research successful and wider recognition on a national and international forum.

Findings indicate that the foundational facilitator for nurses implementing the clinical trial was the effective leadership style adopted by the NL. NLs and role models are crucial in driving research within nursing clinical practice (Akerjordet et al., 2012; Birkhoff et al., 2020; Lode et al., 2015). Participants noted characteristics including optimism, enthusiasm, accessibility and drive to conduct the clinical trial inspired the team to become and stay involved. The NL created an inclusive environment where nurses, regardless of their role or experience, were included. This created a team approach and collective responsibility to empower the nurses to take the project forward themselves (Collins et al., 2020; Ferreira et al., 2020). Nurses cited how the NL's inclusive strategy of involving experienced and recently qualified nurses in the clinical trial created a sense of shared accountability across the team.

The NL guided them in how to function within the research study and the importance of understanding and meeting the clinical trial protocol requirements. Collins et al. (2020) recognize this as developing professional values and responsibilities. Participants became familiar with the defined nature of research, as well as the rigour and transparency required to undertake it successfully. Moreover, the NL created an environment of continual learning (Chen et al., 2019) and embedded an ethos of co‐learning during the clinical trial in which nurses learned from each other. Adopting a train‐the‐trainer approach, junior or new staff members were upskilled by senior or more experienced nurses, meaning that the NL was not responsible for all training. Thus, the team supported each other to address knowledge gaps that arose in real‐time and resolved issues with their peers.

Nevertheless, an important facet cited by nurses was that the NL was accessible to them during the trial, echoing Ferreira et al.'s (2020) findings. While the nurses identified how the NL was available to them for training, troubleshooting and informal conversations about the clinical trial, they also valued the innovative training and supports they implemented, echoing Hudson's (2020) findings. Nurses benefited from a WhatsApp group for learning involving the team and educational posters located in the cancer centre, created by the NL.

Similar to findings by Chen et al. (2019) and Ferreira et al. (2020) nurses in this study appreciated being informed of the trial's progress and outcomes by the NL who adopted constructive criticism and positive reinforcement as a communication style in the process. Nurses felt that they were in a safe space where errors (e.g., in recruitment or data collection) if made, were seen as opportunities for learning. The NL's communication approach demonstrated an effective leadership skill identified in previous studies (Doody & Doody, 2012; Hudson, 2020).

As previously mentioned, while nurses were not exposed to the clinical trial study design or the data analysis, their involvement made them aware of key research components, such as ethics and protocol adherence. Nurses felt their involvement demystified the process of research and consequently they would be more open to engaging with research with the appropriate leader in the future. In this way, it could be argued that the nurses' participation in the clinical trial, was a starting point in empowering future research activity. The learning experience of the clinical trial was a reference point for building research capacity within this nursing team.

## 5 APPLYING TRANSFORMATIONAL LEADERSHIP IN NURSING RESEARCH

The evidence emerging from this study suggests that a leadership style influences followers by building trust, using effective communication skills, encouraging engagement in decision making and empowerment in achieving common goals (Ferreira et al., 2020). More specifically, the style of leadership identified by the nurses is akin to that of transformational leadership (TL) (Collins et al., 2020; Ferreira et al., 2020). The four elements of TL (i.e., ideal influence, inspirational motivation, intellectual stimulation and personal consideration) (Collins et al., 2020; Ferreira et al., 2020; Hudson, 2020; Xu, 2017) were evident in the participants' experience of the research. Transformational leaders can inspire through their innate personality traits and characteristics, such as emotional intelligence (Wang et al., 2018), which cannot be learned. They cultivate relationships based on trust, respect and recognition of individuals' abilities (Hudson, 2020) and foster job satisfaction (Boamah et al., 2018; Wu et al., 2020). TL was effective in empowering nurses in this study to engage in a collective learn‐by‐doing approach to implement the clinical trial. The nursing team took ownership of the clinical trial and recognized their direct contribution to the evidence‐base in line with their increasing sense of empowerment in clinical research activities.

**FIGURE 2:**
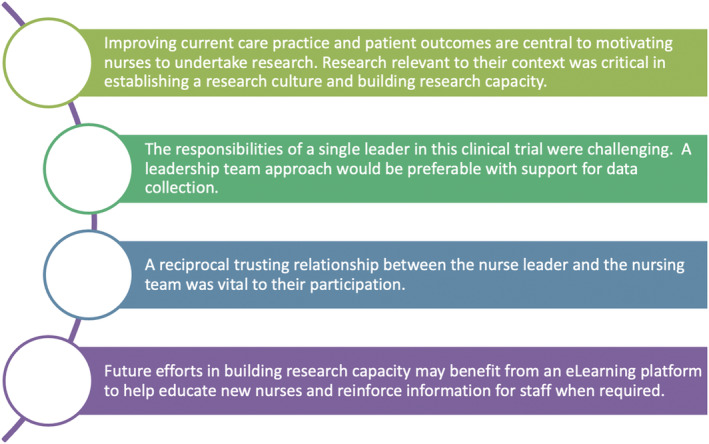
Reflections from the nurse leader

Transformational leaders must have self‐confidence and firmly believe in the vision and have courage to achieve their goals (Xu, 2017) which participants observed through the NL's implementation of and unwavering commitment to the clinical trial for its duration. While TL has been previously discussed regarding clinical practice (Collins et al., 2020; Ferreira et al., 2020; Xu, 2017), to the best of the authors' knowledge, this is the first study to explore its role in building research capacity amongst nurses. Our findings contribute to the existing knowledge regarding transformational leaders being change‐agents in nursing practice (Boamah et al., 2018) by highlighting their significance in implementing and building research capacity. Figure 2 presents reflections of the NL regarding her experience of co‐ordinating the research within clinical practice.

## 6 LIMITATIONS OF THE CURRENT STUDY

A key limitation of this study is the retrospective nature of the data given that it was collected after the clinical trial was completed. A longitudinal approach may have garnered in‐depth findings regarding how nurses' attitudes to and capacity for research changed over time. Additionally, the findings pertain to one clinical setting and the inclusion of other cancer centres may have facilitated a broader understanding of the subject within various settings. Grounded in the participants' accounts, the authors identified transformational leadership as being aligned with their experience. Nevertheless, other leadership approaches may exist that could be applied.

## 7 CONCLUSION

This study highlights to nurse leaders the facilitators of implementing research activities within nursing clinical practice, with a view to building research capacity amongst clinical nurses. Key motivators include the relevance of the research topic, potential to improve patient care and an effective nurse leader. Leaders are change‐agents and can effectively establish a supportive environment in which to implement and conduct research. Positive relationships and trust between leaders and nurses are essential in implementing and undertaking research. The leadership style experienced by the nurses within this study is that of transformational leadership. Through this approach, the nurse leader successfully guided the team to the completion of a clinical trial and ensured the development of transferable research skills, which should help to future‐proof a new generation of nurse researchers.

## 8 IMPLICATIONS FOR NURSING MANAGEMENT

TL is an effective model to empower nurses to implement research within clinical practice and to cultivate a research culture. While nurses may be more likely to engage in research if it leads to improved practice or patient care, consideration also must be given to how the research is implemented and the leadership style employed to engage a nursing team. TL has been found to be crucial when inspiring change, innovation and creativity in practice (Collins et al., 2020; Ferreira et al., 2020; Hudson, 2020). The NL in this study understood the context in which the team worked which facilitated a shared understanding of what it meant to implement the research in their practice. Acknowledging that nurses may not be trained or have experience in clinical research, appropriate resources and ongoing training must be available that is feasible for the clinical setting in which they are working. The findings reflect previous evidence that TL can be overwhelming for one person (Doody & Doody, 2012) and support the adoption of leadership teams while conducting nursing research.

## CONFLICT OF INTEREST

The authors report no actual or potential conflicts of interest.

## ETHICALS STATEMENT

Approval was obtained from the local ethics committee. University College Dublin Research Ethics Committee (Ref. LS‐E‐19‐76‐Darley) and Research & Innovation Committee at the St James's Dublin (Ref. NRAC 192).

## Data Availability

The data that support the findings of this study are available on request from the corresponding author. The data are not publicly available due to privacy or ethical restrictions.
